# Impact of Intravenous Vitamin C Administration in Reducing Severity of Symptoms in Breast Cancer Patients During Treatment

**DOI:** 10.7759/cureus.14867

**Published:** 2021-05-06

**Authors:** Farah Mansoor, Sham Kumar, Prashant Rai, Faryal Anees, Navneet Kaur, Arooj Devi, Besham Kumar, Muhammad Khizar Memon, Sidrah Khan

**Affiliations:** 1 Internal Medicine, Jinnah Postgraduate Medical Centre, Karachi, PAK; 2 Internal Medicine, Civil Hospital Karachi, Karachi, PAK; 3 Obstetrics and Gynecology, Agha Khan University Hospital, Karachi, PAK; 4 Internal Medicine, Adesh Institute of Medical Sciences and Research, Buchu Kalan, IND; 5 Oncology, Ghulam Muhammad Mahar Medical College, Sukkur, PAK; 6 Internal Medicine, Liaquat University of Medical and Health Sciences, Hyderabad, PAK

**Keywords:** breast cancer, complementary medicine, vitamin c, intravenous vitamin c, pakistan

## Abstract

Introduction

Alternative medicine during treatment is often used to make the quality of life (QoL) better. Women with early-stage breast cancer, particularly the ones who possess lower QoL, are more prone to opt for complementary medicine. This study aims to explore the effects exerted by intravenous vitamin C (IVC) on symptoms and adverse events associated with breast cancer treatment.

Methods

This single-center, parallel-group, single-blind interventional study was conducted in the oncology ward of a tertiary care hospital in Pakistan. For this study, after informed consent was taken, breast cancer patients with Union for International Cancer Control stages IIA to IIIb were included in the study. Three hundred and fifty (n = 350) patients were randomized into two groups at a ratio of 1:1. Study group was randomized to receive 25 grams per week of IVC at a rate of 15 grams per hour for four weeks in addition to their current standard treatment, and the control group received placebo (normal saline drip with label removed) in addition to their current standard treatment.

Results

In patients who had received IVC, there was a significant decrease in the mean severity score after 28 days for the following symptoms: nausea (2.65 ± 0.62 vs. 2.59 ± 0.68; p-value: 0.0003), loss of appetite (2.26 ± 0.51 vs. 2.11 ± 0.52; p-value: 0.007), tumor pain (2.22 ± 0.45 vs. 1.99 ± 0.40, p-value: <0.0001), fatigue (3.11 ± 0.32 vs. 2.87 ± 0.29; p-value: <0.0001), and insomnia (2.59 ± 0.35 vs. 2.32 ± 0.36, p-value: <0.0001).

Conclusion

Our study showed improvement in the mean severity score of nausea, fatigue, tumor pain, loss of appetite, and fatigue. More studies are also needed to assess the long-term effects of IVC in the cancer management. This shall help incorporate the use of IVC in standard practice to make the journey of cancer management comfortable for the patients.

## Introduction

Breast cancer is the most common malignancy found in women worldwide [1]. Among all the cancer cases reported around the world, breast cancer makes up 23% of all cases. In Pakistan, there is a high prevalence of breast cancer, with around 90,000 cases being diagnosed annually and every 1/9 woman affected [2]. Most of the patients present with a breast lump, nipple discharge, dimpling of skin, and change in the size of the breast and/or an eczematous patch. Patients who present with advanced disease may present with symptoms of metastatic disease like bone pain, dyspnea, jaundice, and lymphadenopathy [3]. Treatment of breast cancer is associated with various common side effects including nausea, vomiting, pain, fatigue, insomnia, and depression [4]. Due to the treatment-associated adverse effects, quality of life (QoL) is considered as a well-accepted outcome measure for cancer patients [5]. Decrease in the health-related QoL secondary to the side effects of chemotherapy may result in early discontinuation of treatment in patients [6].

Alternative medicine during treatment is often used to make the QoL better. Women with early-stage breast cancer, particularly the ones who possess lower QoL, are more prone to opt for complementary medicine [7]. The American Cancer Society has stated that complementary medicine or methods are those that are taken along with the standard treatment regimen. If adopted and controlled with precautions, they could potentially result in causing ease and betterment [8]. There are several treatment options available for breast cancer patients. One of the modalities proposed to supplement the treatment is high-dose intravenous vitamin C (IVC). It has demonstrated improvement in the QoL by decreasing several distressful symptoms, including pain and fatigue. In a study, patients receiving high-dose IVC, evaluated at intervals of two and four weeks, reported 46.7% and 60% improvement in QOL, respectively [9]. This study aims to explore the effects exerted by IVC in breast cancer patients during their treatment. If proven effective, it will provide an option of low-cost complementary medicine to reduce the adverse events associated with chemotherapy and radiotherapy and improve QoL in patients with breast cancer.

## Materials and methods

This single-center, parallel-group, single-blind interventional study was conducted in the oncology ward of a tertiary care hospital in Pakistan from March 2019 to November 2019. For this study, after informed consent was taken, breast cancer patients with Union for International Cancer Control (UICC) stages IIA to IIIb were included in the study. Patients in stage I were not included, as surgery is the main treatment for them. Patients with stage IV cancer were not enrolled, as most patients were under palliative care. Patients were enrolled using consecutive convenient non-probability sampling.

At the time of study, 441 patients were undergoing treatment for breast cancer in the oncology ward, of which 371 were eligible for study. Twenty-one patients refused to give consent. Three hundred and fifty patients were randomized into two groups using online randomizer software Research Randomizer (https://www.randomizer.org/) at a ratio of 1:1. The study group was randomized to receive 25 grams per week of IVC at a rate of 15 grams per hour for four weeks in addition to their current standard treatment, and the control group received placebo (normal saline drip with label removed) in addition to their current standard treatment. Standard treatment included chemotherapy, radiotherapy, and hormone therapy.

After enrollment and registration, patient’s demographics, grade of cancer, and current treatment were noted in a self-structured questionnaire. The severity of the following symptoms were recorded using a visual analog scale (VAS): diarrhea, nausea, loss of appetite, vomiting, tumor pain, fatigue, and sleep disorders. VAS had a reading of zero to four; zero means no symptoms while four means very severe symptoms. Questionnaire was explained to participants and participants were asked to mark the most suitable option for each symptom. Participants were followed for four weeks, and at the end of follow-up, severity of symptoms were again noted via VAS. During follow-up, two patients were lost from each group. One patient died in the study group and two patients died in the control group. One hundred and seventy-two participants completed the follow-up in the study group and 171 participants completed the follow-up in the control group. Only participants who completed the study were included in the final analysis.

Statistical analysis was done using the Statistical Package for the Social Sciences (SPSS v. 23.0) (IBM Corporation, Armonk, New York, USA). Numerical data were presented as mean and standard deviation, while categorical data were presented as frequency and percentages. Mean values at day 0 and day 28 for both groups were compared using dependent t-test. P-value of less than 0.05 meant that the difference between intervention and control group is significant and null hypothesis is not valid.

## Results

Mean age of participants in the study and the control group was 57 ± 9 years and 58 ± 9 years, respectively. Both groups were also comparable in terms of stage of cancer and treatment (Table 1).

**Table 1:** A comparative analysis of both groups based on cancer staging and treatment

Characteristics	Study Group (n = 172)	Control Group (n = 171)	p-Value
Mean age (years)	57 ± 9	58 ± 9	0.3
Union for International Cancer Control (UICC) stages			
IIa	98 (56.98%)	91 (53.22%)	0.87
IIb	35 (20.35%)	40 (23.29%)	
IIIb	27 (15.70%)	29 (19.96%)	
IIIb	12 (6.98%)	11 (6.43%)	
Treatment			
Chemotherapy	131 (76.16%)	127 (74.27%)	0.68
Radiotherapy	51 (29.65%)	48 (28.07%)	0.74
Hormone therapy	32 (18.60%)	36 (21.05%)	0.56

In patients who had received IVC, there was a significant decrease in mean severity score after 28 days for the following symptoms: nausea (2.65 ± 0.62 vs. 2.59 ± 0.68; p-value: 0.0003), loss of appetite (2.26 ± 0.51 vs. 2.11 ± 0.52; p-value: 0.007), tumor pain (2.22 ± 0.45 vs. 1.99 ± 0.40, p-value: <0.0001*), fatigue (3.11 ± 0.32 vs. 2.87 ± 0.29; p-value: <0.0001*), and insomnia (2.59 ± 0.35 vs. 2.32 ± 0.36, p-value: <0.0001). There was no difference in mean severity score of any symptom in the placebo group (Table 2).

**Table 2:** Analysis of change in severity of symptoms after IVC treatment using VAS score IVC, intravenous vitamin C; VAS, visual analog scale *: Significant

Symptoms	Study Group			Control Group		
	Day 0 Mean VAS Score	Day 28 Mean VAS Score	p-Value	Day 0 Mean VAS Score	Day 28 Mean VAS Score	p-Value
Diarrhea	2.65 ± 0.62	2.59 ± 0.68	0.39	2.51 ± 0.55	2.58 ± 0.50	0.2
Nausea	3.01 ± 0.62	2.78 ± 0.54	0.0003*	2.98 ± 0.55	2.92 ± 0.52	0.3
Loss of appetite	2.26 ± 0.51	2.11 ± 0.52	0.007*	2.41 ± 0.62	2.47 ± 0.54	0.34
Vomiting	2.87 ± 0.56	2.77 ± 0.50	0.08	2.81 ± 0.41	2.86 ± 0.34	0.22
Tumor pain	2.22 ± 0.45	1.99 ± 0.40	<0.0001*	2.31 ± 0.47	2.40 ± 0.46	0.07
Fatigue	3.11 ± 0.32	2.87 ± 0.29	<0.0001*	3.08 ± 0.40	3.09 ± 0.44	0.82
Insomnia	2.59 ± 0.35	2.32 ± 0.36	<0.0001*	2.62 ± 0.42	2.59 ± 0.44	0.51

## Discussion

Although the role of vitamin C and other antioxidants as a complementary treatment in cancer management has been of keen interest for scientists, limited literature exists on its practical use and clinical effects in humans, particularly in those with breast cancer [10-12]. Our study aimed to fill this gap in knowledge and tried to add to the scarce evidence pool by exploring the role of IVC in improving QoL in patients with breast cancer. We found that vitamin C plays a beneficial role in alleviating symptoms arising either as side effects of standard adjuvant therapy or by cancer itself. In our sample, most patients belonged to UICC stage IIa for breast cancer with almost 75% of patients undergoing chemotherapy. The randomization led to no significant difference in baseline characteristics of patients receiving IVC and those in the control group. Results show that per-week administration of 25 grams of IVC improved mean VAS scores for all the symptoms at 28 days post vitamin C initiation. However, significant improvement was notably seen in symptoms pertaining to the gastrointestinal tract, such as nausea and appetite, systemic and neural symptoms like tumor pain, fatigue, and insomnia. The patients in the treatment group reported no new side effects after initiation of IVC.

The current study findings seem to be consistent with the results of a handful of studies published previously on the topic [12-15]. A study conducted in Korea in terminally ill cancer patients (including breast cancer) reported that IVC significantly improved physical, emotional, and cognitive function as well as ameliorated the symptoms of nausea, vomiting, and fatigue and improved the appetite [13]. Similar results were found in a German study conducted in breast cancer patients where administration of 7.5 grams of IVC in patients significantly improved nausea, loss of appetite, fatigue, dizziness, sleep disorders, depression, and hemorrhagic diathesis [14]. Consistent findings were also seen in a case report of a woman with recurrent breast cancer receiving once-weekly chemotherapy. It was found that twice-weekly administration of 50 grams of vitamin C dramatically decreased fatigue and insomnia while concomitantly improving cognitive functioning [15]. Compared to our study, none of these studies reported any side effects of the IV administration of vitamin C. Despite the difference in the doses of vitamin C in the different studies, the results remained consistently positive and endorsed the role of vitamin C administration for alleviation of symptoms in breast cancer patients. 

The role of vitamin C in reducing symptoms in cancer patients can be explained by its antioxidant properties. It is known that radiation and chemotherapy along with tumor cell metabolism increase oxidative stress in cancer patients [14-18]. This stress is combated by the intrinsic antioxidants of the body including vitamin C [14]. It is seen that patients with cancer have low levels of vitamin C in their bodies [19]. This is because uncontrolled oxidative stress in cancer leads to high consumption of intrinsic body reserves resulting in vitamin C depletion. If not replenished duly, this deficiency eventually leads to the unopposed production of reactive oxygen species (ROS) [20,21]. The gut mucosa and the neural tissues, being the most sensitive to ROS, are affected the most. This leads to mucosal irritation of the gastrointestinal tract causing symptoms of nausea, vomiting, and loss of appetite. The neural irritation may trigger mental disorders including insomnia, tumor pain, and fatigue [14,22,23]. Thus, replenishment of the intrinsic reserve by parenteral vitamin C administration may help combat the unopposed ROS production and play a vital role in alleviating cancer symptomatology, and hence QoL in these patients, as witnessed in our current study.

Our study has a few limitations that must be highlighted. Due to lack of resources and difficulty in maintaining follow-up, our study assessed only the short-term effects of IVC on QoL in breast cancer patients. Although convenient sampling was employed for participant recruitment, patient allocation to the treatment and control groups was randomized to minimize the risk of selection bias. Most participants were elderly and belonged to the UICC stage IIa; therefore, the generalization of the current findings to all breast cancer patients must be done cautiously. Despite the limitations, our study adds to the limited literature on the role of parenteral vitamin C in breast cancer patients. As the intervention was physician-led, it dodges the concerns of patient adherence to the treatment and its confounding effect on the study outcomes. The use of 25 grams of vitamin C in the present study is unique and never been used before in the other trials hence providing evidence in support of a range of doses without the consequence of side effects [13-15].

## Conclusions

Our study showed improvement in the mean severity score of nausea, fatigue, tumor pain, loss of appetite, and fatigue. Although our findings suggest that vitamin C can significantly improve QoL in breast cancer patients, more studies with a larger sample size and use of different vitamin C doses are needed to confidently infer the findings to all breast cancer patients and to determine the most potent dose, respectively. More studies are also needed to assess the long-term effects of vitamin C in cancer management. This shall help incorporate the use of vitamin C in standard practice to make the journey of cancer management comfortable for the patients.
